# A consideration on the one-part mixing method of alkali-activated material: problems of sodium silicate solubility and quick setting

**DOI:** 10.1016/j.heliyon.2022.e08783

**Published:** 2022-01-17

**Authors:** Mohammad Idris Rasuli, Yuyun Tajunnisa, Akifumi Yamamura, Mitsuhiro Shigeishi

**Affiliations:** a Graduate School of Science and Technology, Kumamoto University, 2-39-1 Kurokami, Chuo-ku, Kumamoto, 860-8555, Japan; b Institut Teknologi Sepuluh Nopember (ITS), Kampus ITS Manyar, Jl. Menur 127, Surabaya, 60116, Indonesia; c Nippon Koei Co., Ltd., 5-4 Kojimachi, Chiyoda-ku, Tokyo, 102-8539, Japan; d Faculty of Advanced Science and Technology, Kumamoto University, 2-39-1 Kurokami, Chuo-ku, Kumamoto, 860-8555, Japan

**Keywords:** Alkali-activated material, Sodium metasilicate, Solubility, Sucrose, Setting time test

## Abstract

This research investigates the properties of alkali-activated materials (AAMs) using sodium metasilicate, with the ratio of SiO_2_:Na_2_O equals 1. This study was conducted to achieve the following three aims. Firstly, to understand the solubility mechanism of granular sodium metasilicate pentahydrate (Na_2_SiO_3_.5H_2_O) when used in a one-part mixing method. Secondly, to investigate the properties of AAMs when a sodium metasilicate aqueous solution is used as an alkaline material and as a source of silica. Lastly, to study the retardation effect of sucrose on AAMs. This research used aluminum silicate precursors, such as low-calcium fly ash, slag, and micros silica, alkali activators, such as NaOH pellets and Na_2_SiO_3_.5H_2_O, and standardized sand. The alkaline activators were first dissolved in water using a water bath shaker to achieve the alkaline solution. Sucrose, which is about 2% of the weight of the solid precursors, was added to modify the reaction process between the precursors and the alkaline materials. Four types of samples were prepared: M1, M2, M3, and M4, with the fly ash, slag, and silica fume ratios of 80:20:0, 70:30:0, 75:20:5, and 100:0:0, respectively. The research conducted solubility test of the alkaline materials, flowability, 7-, 28-, 56-day compressive and flexural tests, drying shrinkage test of mortar samples, and the setting tests of pastes with and without sucrose. The results show that the dissolution time of the NaOH was much shorter, whereas Na_2_SiO_3_.5H_2_O needed a solvent with a temperature of around 40 °C to be fully dissolved. This problem of solubility decreases the quality of AAMs formed using the one-part mixing method. Among the mortar samples, the M4 had the highest flow rate, while M3 had the lowest flow rate. M2 had the highest compressive and flexural strength of 43.4 MPa and 6.1 MPa, respectively. The setting time test shows that sucrose retards the reaction process in AAM.

## 1 Introduction

Portland cement concrete is the most commonly used construction material in the world. Annual global production of ordinary Portland cement (OPC) reaches 4 billion tons [1]. It is not environmentally friendly as it is responsible for 8% of global CO_2_ emissions [2]. Moreover, byproducts of some materials have a negative influence on the environment of the world. Coal-based power plants burn more than 8 billion tons of coal every year [3]. The production of unused fly and bottom ashes from coal-based power plants is mainly disposed of and negatively influences the environment and groundwater quality due to the metals leaching from the ashes [4].

When aluminosilicate materials, such as fly ash and slag, react with an alkali source, it produces a material that has binding properties [5, 6]. Such materials are called geopolymers or alkali-activated materials (AAMs). Compared with OPC, AAMs are more environmentally friendly because they emit a much lower amount of CO_2_ to the atmosphere [7, 8]. Moreover, they contain some byproducts as their primary constituents, such as fly ash and slags [9]. Besides being environmentally friendly, AAMs have some other advantages, such as obtaining high early strength and superior mechanical properties [10, 11]. Furthermore, they have better resistance to fire, acid attack, and alkali-silica reaction [12, 13, 14].

Aqueous alkaline solution, such as NaOH, KOH, Na_2_SiO_3_, and K_2_SiO_3_, is used as an alkali source in AAMs, as geopolymerization and activation of AAM occur in an alkaline environment. Water glass (Na_2_SiO_3_) provides such an environment [15], and they are a source of silica for the synthesis of the binders [16]. Moreover, only alkaline hydroxide activators will result in low-rate reactions compared to those containing soluble silicate [17], and sodium silicate increases the reaction process between alkali and aluminosilicate precursors [18]. Some studies have used other metal silicates instead of Na_2_SiO_3_, such as potassium silicate. However, using potassium silicate has some disadvantages, such as increased specific surface area values, a lower degree of crystallinity, and lower resistance to attack by HCL [16]. Therefore, the vast majority of water-soluble alkali silicates used in geopolymerization is sodium silicates because they are cheaper and produced in substantially larger quantities than potassium silicates [6].

AAMs are made conventionally by the reaction of aluminosilicate precursors, such as fly ash and slag, and aqueous alkaline solution, such as hydroxides, silicate, and carbonate. This method for making the materials is called the two-part mixing method [19, 20, 21]. Handling and working with corrosive and viscous alkaline solutions causes difficulties in their application, and they require special skill and caution. Therefore, this requirement limits the material's production in factories for precast purposes [22]. One-part mixing method or dry mixing method is being studied by some researchers [22, 23, 24] in which aluminosilicate precursors and alkali materials are used in solid-state, and water is added just for their reaction purpose. Cast-in situ or cast-in-place and secure application of the materials are the main aims of studying this method [22].

The author of this study investigated the properties of AAM made by both one-part and two-part mixing methods and compared both results in previous unpublished research. Sodium hydroxide (NaOH) pellets and sodium metasilicate pentahydrate (Na_2_SiO_3_.5H_2_O), a type of sodium metasilicate, were used as alkaline activator (AA). The result showed that the strength of AAMs made using the one-part mixing method was far less than the one made by the two-part mixing method. Moreover, some other researchers have also reported that the AAMs made using the one-part mixing method had lower mechanical strength than the one made using the two-part mixing method [25, 26]. Furthermore, Ouyang et al [25] found some undissolved particles of sodium metasilicate pentahydrate by studying the microstructure of the samples. Therefore, inappropriate dissolution of the alkaline source might have caused the problem. The alkaline solution is the main part of geopolymers and AAMs for geopolymerization and activation of the aluminosilicate precursors to make the binder [27]. Hence, there is a need for studying the solubility of both kinds of alkaline activators: NaOH pellets and granular Na_2_SiO_3_.5H_2_O in different solvent temperatures. The result will help the further development of the method.

The granular Na_2_SiO_3_.5H_2_O was used in the author's previous experiment for making AAMs using the one-part mixing method. Moreover, some other researchers used the same material while adopting the same mixing method [22, 25, 28, 29]. The extensive use of the material may be due to its higher solubility [30, 31] and availability compared to the other types of solid sodium silicate. So, there is a need to study the properties of sodium metasilicate aqueous solution and its influence on the characteristics of AAMs. Currently, there is not any study available regarding that. Moreover, this experiment characterizes the applicability of sodium metasilicate solution in a two-part mixing method. Traditionally, in a two-part mixing method, AAMs are produced using a Na_2_SiO_3_ solution that has a modulus (SiO_2_:Na_2_O) of around 2, which makes the AAM viscose and causes application problems [32, 33, 34]. Besides, sodium metasilicate has a modulus of 1. According to Davidovits [35], the viscosity of the Na_2_SiO_3_ is decreased when reducing the SiO_2_:Na_2_O ratio.

Fly ash-based AAMs, especially those with a small amount of Ca+ in their composition, have a much higher setting time due to their slow reactivity. Therefore, they are cured at higher temperatures [36, 37]. Incorporating slag with fly ash increases the reactivity of aluminum silicate precursors that results in quick hardening of the samples at ambient temperature and modifies other properties of concrete, such as compressive strength, flexural strength, and density of concrete [38]. As the slag is very active and increases the reactivity and hydration of the materials [39], its addition causes quick solidification of the material that in some cases causes difficulty in casting the fresh AAM in molds.

The authors observed the problem of quick setting in a one-part mixed AAM mortar, which had a significant amount of slag in their composition in the previous unpublished experiment. Using sucrose delayed the solidification of the mortar, thereby making ease in the casting of the mortar. Furthermore, sucrose increased the strength of the mortar. This may be due to the modification of the reaction process caused by the addition of sucrose and better dispersion of the precursors and alkaline material throughout the material because of the improved workability. This research also studies the retardation behavior of sucrose on AAMs through setting time test since the retarding behavior of sucrose on OPC materials is reported by some researchers. Some researchers have concluded that sucrose retards the setting time and improves the microstructure of OPC concrete [40, 41]. Besides sucrose, some other kinds of retarder are also available, such as lignosulfonic acid, hydroxycarboxylic acids, and their salts, and carbohydrates, such as glucose [42]. But using sucrose as a retarder has the advantage of sustainability and availability.

## 2 Experiments

### 2.1 Materials

Fly ash type II, ground granulated blast-furnace slag (GGBFS) with a Blaine size of 6000 cm^2^/gr, and microsilica were used as aluminum silicate precursors. Standardized sand compatible with the ISO 679:2009 was used as fine aggregate, and the sand to binder ratio was 2.97. The chemical composition of the materials is shown in Table 1. The alkaline materials used were NaOH pellets (98% purity) and granular Na_2_SiO_3_.5H_2_O that consists of 30% Na_2_O, 29% SiO_2_, and 41% H_2_O. Sucrose, which is about 2% of the weight of the solid precursors, was added to modify the reaction process between aluminosilicate precursors and alkaline materials.

**Table 1:** Specific gravity (S.G.), the chemical composition of materials in weight percentage (wt. %) by XRF, and chemical analysis of standardized sand.

Materials	Chemical composition (% weight)							S.G.(g/cc)
	SiO_2_	Al_2_O_3_	Fe_2_O_3_	CaO	Na_2_O	H_2_O	K_2_O	
Fly ash	55.19	25.35	7.57	4.06				2.33
GGBFS	35	16	0.7	46				2.89
Microsilica	93.67	0.83	1.3	0.31	0.4			2.22
Standardized sand	98.4	0.41	0.36	0.16	0.01	<0.2	0.01	2.64

Overall, four types of mortar samples were made in this experiment. Tables 2 and 3 show the percentages of aluminosilicate precursors and the mixture proportion of each sample, respectively. NaOH solution's molarity was 8 M. The concentration of the sodium metasilicate solution was set to 32%, and the ratio of the alkaline solutions (Na_2_SiO_3_: NaOH) was 1.5 for all types of mortar and paste samples. The liquid to binder ratio was set to 0.33.

**Table 2:** Percentages of the aluminosilicate precursors in each sample.

Sample	Fly ash (%)	GGBFS (%)	Silica fume (%)
M1	80	20	0
M2	70	30	0
M3	75	20	5
M4	100	0	0

**Table 3:** Mixture proportions of AAM mortar (kg/m^3^).

Mix	Aluminosilicate materials			Sand	Alkali activator (AA) aqueous solution			Mixing ​Water (10% of aluminosilicate materials)	Sucrose	L/B∗
	Fly ash	GGBFS	Microsilica		NaOH	Na_2_SiO_3_.5H_2_O	Solvent water			
M1	422.8	105.7	0	1536.2	29.8	93.9	160.8	52.9	10.6	0.33
M2	373.9	160.2	0	1536.2	31.0	97.2	166.4	53.5	10.7	0.33
M3	397.9	106.1	26.5	1536.2	29.8	93.9	160.8	53.0	10.6	0.33
M4	517.6	0.0	0	1536.2	29.2	92.1	157.5	51.8	10.4	0.33

∗ Liquid/binder. L: Solvent water + Mixing water (10% of aluminosilicate precursors). Binder: Aluminosilicate materials + NaOH + Na_2_SiO_3_.5H_2_O.

### 2.2 Preparation of the materials

#### 2.2.1 Premixing the precursors

To improve the reaction process of making the AAM, fly ash, slag, and silica were premixed in a mixer called Omni mixer.

An Omni mixer with a 5 L capacity and rotation speed of 2.5 Hz (2.5 spin or round per second) was used to mix the materials. The mixing duration was first set to 2 h. However, when the materials were not appropriately mixed and some white slag particles were visible, the mixing duration was increased to 3 h. After the 3-hour mixing, the materials were well mixed than before. The premixed materials were covered by plastic bags and put inside a bucket with a lid. The bucket was then covered with a lid.

#### 2.2.2 Making alkali solution

NaOH pellets can quickly dissolve in water at room temperature, but granular Na_2_SiO_3_.5H_2_O cannot easily dissolve in water at room temperature and need a solvent with a temperature of around 40 °C or above. This was found by the solubility test of the alkaline materials conducted in this study. The details and results of the experiment are explained in the result section.

A water bath shaker was used to make an AA solution. It was equipped with a heater to control the temperature of the water inside, wires to hold the flasks, and a shaking mechanism. The making of the alkaline solution was as follows. First, the shaker's tub was half-filled with water. Then, flasks with a specified amount of distilled water were placed inside it and tightened by its wires. The temperature of the water inside the tub was set to 30 °C. The shaking rate was 120 rounds/min. A specific NaOH powder was put inside each flask in two sets, then shook for 3 min for complete dissolution. Granular Na_2_SiO_3_.5H_2_O was placed inside the flasks in 3 equal groups of shaking. During the dissolution of NaOH pellets in water, the solution's temperature increased. It helped the first group of granular Na_2_SiO_3_.H_2_O to be well dissolved in the NaOH solution. When the temperature decreased, the granular Na_2_SiO_3_.5H_2_O could not be dissolved well. Therefore, in the second and third sets, the tub water temperature was increased to 40°C–45 °C, respectively.

After Na_2_SiO_3_.5H_2_O dissolved entirely in NaOH solution, it was put in a larger flask and stored at room temperature. After some time, it was noticed that the solution was susceptible at normal temperature, and solution crystallization could quickly occur inside the solution. Therefore, the AA solution was kept in an incubator at 45 °C immediately after its making. After that, the crystallization did not occur, and the solution was transparent, smooth, not viscose, and looked like water.

### 2.3 Mixing, casting, and curing

The experiment prepared prisms of 160 × 40 × 40 mm for both compressive and flexural strength and shrinkage tests. The materials were mixed in a paddle mixer.

During the mixing procedure, as the solution's temperature decreased due to the solute crystallization, the flow and workability of the mortars decreased. Therefore, it was too difficult to cast them into molds. This mixing condition was called the ambient mixing condition.

Then, it was decided to keep the mixing materials in the incubator at 45 °C for at least 24 h, and then mix the materials at a constant temperature of 24°C–26 °C. This mixing condition was named the hot mixing condition. The mixing procedure was done inside a tent at above mentioned temperature. The study observed a significant improvement in the flow and rheology of the mortar.

The flowability was measured according to JIS R5201. A flow table was used to test the flowability. The mortar was put in the cone in two equal layers. Each layer was tamped 15 times by a tamped rod. Then, excess mortar material was cut off and the surface of the cone was flattened. Then, the cone was slowly lifted upward. The plate was then repeatedly risen and dropped 15 times. Next, the mortar was spread. The maximum direction and the opposed direction, which was perpendicular to the maximum direction were measured. This procedure was done twice and the average value in millimeter (mm) was taken as the flow value of the mortar. After that, the mixtures were poured inside prisms and vibrated for 2 min. To avoid crystallization of the solutes inside the mix and facilitate the reaction process between aluminum silicate precursors and alkaline solution, the prisms were put at 45 °C and 94% relative humidity inside an incubator for two days. After that, they were cured in the control room at 18°C–20 °C and 30%–50% relative humidity.

In this research, the solubility test of the solid alkaline materials, flow test, 7-, 28-, 56-day compressive and flexural strength test, and drying shrinkage of mortar samples were studied. Moreover, to investigate the retarding effect of sucrose on AAM, the mixed proportion of the raw precursors of M2, which has the highest amount of slag, and M4 were chosen to make pastes for studying the initial and final setting time. The pastes were studied in the presence and absence of 2% sucrose.

## 3 Results and discussion

### 3.1 Solubility of the solid alkaline materials

The reaction between aluminosilicate precursors and alkaline materials is vital for producing geopolymers and AAMs. Therefore, in a one-part mixing method, solid alkaline materials, such as NaOH and sodium metasilicate need to be well dissolved to produce the desired AAM.

A solubility test of the materials was conducted to better understand the alkaline materials’ solubility mechanism in the one-part mixing method. One batch mixing amount of NaOH pellets, granular Na_2_SiO_3_.5H_2_O, and water was put inside a flask. The Na_2_SiO_3_.5H_2_O: NaOH ratio was set to 1.5. A water bath shaker was used to mix the materials. The shaking rate was set to 120 rounds/min.

The temperature of the water before mixing with the alkaline materials was 26 °C. NaOH pellets and granular Na_2_SiO_3_.5H_2_O were put inside the flask and shook for 5 min. The duration of shaking was chosen according to the estimated mixing timing of making mortar or concrete in a mixer. After 5 min of mixing, the temperature of the solution increased to about 34 °C. As the dissolution of NaOH in water is an exothermic reaction, it rose the temperature of the solution to about 8 degrees Celsius. Most particles could not be dissolved in water, and those particles were the sodium metasilicate since NaOH quickly dissolved in water. For confirmation, a similar experiment was performed, in which it took less than 5 min for the NaOH to be fully dissolved in water.

Therefore, the solution was mixed for two more minutes, but there was still no improvement observed in the dissolution rate, and the temperature of the solution decreased to 31 °C. Therefore, to increase the temperature of the solution, the temperature of the shaker's tub water was set to 35 °C and then to 40 °C, and each was mixed for 5 min. Thus, the temperature of the solution increased to 32 °C and 36.5 °C, respectively, and a significant improvement in the dissolution of the sodium metasilicate was observed. However, still some particles remained undissolved. When the temperature of the solution increased to 41 °C, total dissolution of the particles occurred.

This study shows that the dissolution of the granular Na_2_SiO_3_.5H_2_O by a solvent with a lower temperature is difficult, and it could not be fully dissolved when adopting the one-part or the drying mixing method to make geopolymers and AAMs. For making 32% sodium metasilicate solution, a temperature of around 40 °C is required for the solvent to dissolve the solid material thoroughly. Concerning increment of some degrees of temperature by NaOH, a solvent with a temperature slightly lower than 40 °C can also be enough for the solubility of the sodium metasilicate. The temperature of the aluminum silicate precursors and aggregates may also influence the solubility of the materials. Therefore, the hot mixing condition will have a significant effect on the one-part mixing method. Moreover, besides maintaining the proposed temperature, increasing the mixing duration and reducing the Na_2_SiO_3_.5H_2_O: NaOH ratio may also play a significant part in the dissolution of the material in the one-part mixing method.

### 3.2 Flowability

The flowability test was conducted to observe the influence of the aluminosilicate precursors and the change in the mixing conditions on the flow of the samples. Moreover, the flow and rheology of the alkaline solution formed by the sodium metasilicate are discussed in this section.

The alkaline solution formed by NaOH pellets and granular Na_2_SiO_3_.5H_2_O was smooth and fluent and there was no sign of viscosity. But as mentioned earlier, the alkali solution was very sensitive to the temperature variation as the crystallization of the AA solution occurred at a temperature lower than 30 °C. Therefore, the workability and flowability of the mortars were decreased at the aforementioned temperature. The same phenomena of crystallization of the water glass or sodium silicate can also happen while adopting the one-part mixing method using granular Na_2_SiO_3_.5H_2_O. The crystallization in the solution may reduce the reaction process between the alkali and the aluminosilicate precursors. Hence, the properties of the AAMs formed can be reduced.

In this study, it was decided to change the temperature of the materials and the mixing condition. All materials were kept at 45 °C for 24 h, and mixing was done at a controlled temperature of 24°C–26 °C. When the mixing condition was changed to the hot mixing condition, significant improvement in the workability of fresh mortar was observed and crystallization did not occur.

Before applying the hot mixing condition, the M1 sample was mixed in an ambient mixing condition, and the flow of the sample was so low that it was not easily put inside the molds. The flow value of the M1 was 102.5 mm. There was about a 15% increment in the flow value of the mixture when the hot mixing condition was applied. A higher flow value was achieved due to a reduction in the mix's cohesiveness at a higher temperature.

**Figure 1:**
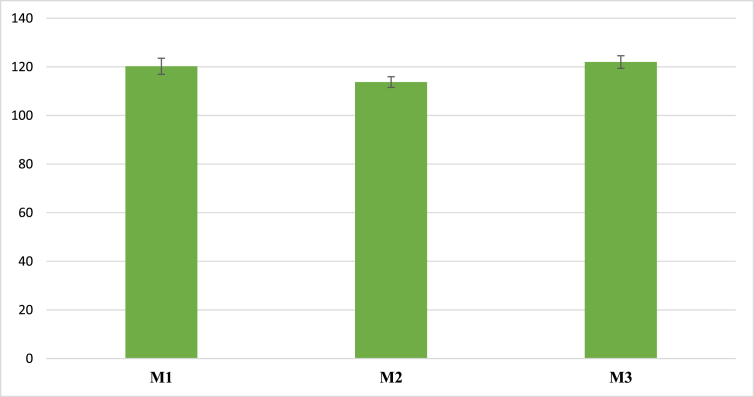
Flow value (mm).

Among the samples, the M4 sample had the highest flowability, and its flow value could not be measured due to its overflow from the flow table. It was due to the low reactivity of low-calcium fly ash or fly ash type II [37] which caused the extremely low chemical reaction rate between the precursor and the alkaline solution, therefore, a lower amount of the aqueous solution was consumed during the reaction process. There was a decrease in the flowability of slag and microsilica due to their higher reactivity [38, 39]. The mortar with 30% of slag had the lowest flow value of 114 mm M3 and M1 had a flowability of 122 and 120 mm, respectively. Figure 1 shows the flow value of the mortars.

Previous studies [32, 33, 34, 43] showed high viscosity and low workability for fly ash-based geopolymer using Na-based silicate as an activator. They also reported that admixtures, such as superplasticizers, did not significantly improve the flowability and rheology of paste or mortar. However, this experiment shows that all fresh mortars were smooth, fluent, and without viscosity. This outcome was achieved due to the use of sodium metasilicate and the hot mixing conditions.

### 3.3 Compressive and flexural strength

**Figure 2:**
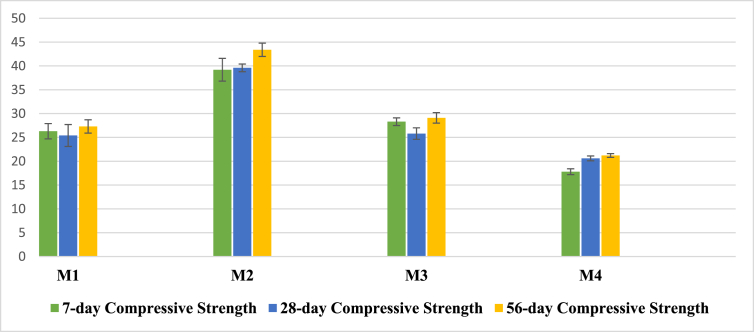
The compressive strength (MPa).

**Figure 3:**
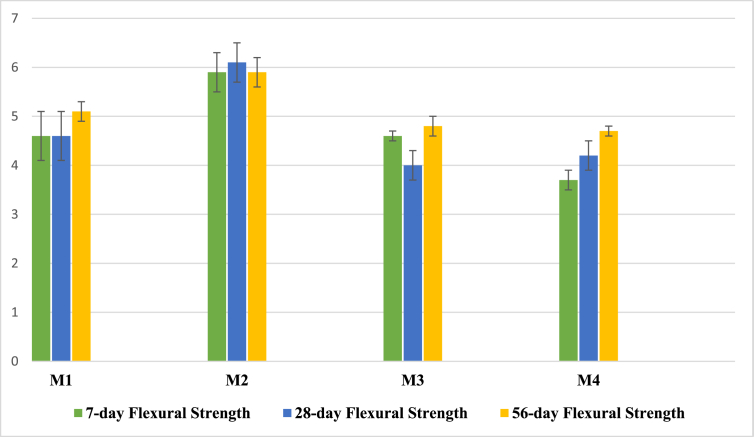
The flexural strength (MPa).

Compressive and flexural strength are an important characterization of AAM. As the sodium silicate improves the reaction process between the precursors and alkali [17], so the compressive and flexural results will reveal if a reasonable strength is achieved by the sodium metasilicate. Therefore, each sample type was tested for compressive and flexural strength at 7, 28, and 56 days. The test used a Universal Testing Machine with a 200 tons capacity. Figures 2 and 3 show the compressive and flexural values of each type of sample, respectively.

Slag had a significant influence on both the compressive and flexural strength of the samples due to the presence of a high amount of Ca + that increases the reactivity of the material [39]. The M2 sample, made of 30% slag, had the highest compressive strength of 39.2, 39.6, and 43.4 MPa at the age of 7, 28, and 56 days, respectively, followed by M3 and M1.

The M4 sample, made of 100% fly ash, had the lowest compressive strength of 17.8, 20.6, and 21.2 MPa at the ages of 7, 28, and 56 days, respectively. The lowest compressive strength for all ages was due to the extremely low reaction between the aluminosilicate precursor and alkaline solution.

Following the compressive strength result, the M2 sample had the highest 7-, 28-, and 56-day flexural strength, which is approximately 6 MPa for all ages, followed by the M1 and M3 samples, which both had the flexural strength of approximately 5 MPa for all ages. The M4 sample had a flexural strength of 3.7, 4.2, and 4.7 MPa at the ages of 7, 28, and 56 days, respectively.

All samples were cured at 45 °C and 94% relative humidity inside an incubator for two days. Afterward, they were cured at 20 °C and 30%–50% relative humidity. Therefore, they gain most of the strength within a short time. The buildup in strength was due to the increase in the reaction or hydration rate due to heat and steam inside an incubator [44, 45, 46]. The reduction in the 28-day compressive strength of M2 and 28-day compressive and flexural strength of M3 is attributed to the loss of mesopore water during curing at 18°C–20 °C and 30%–50% relative humidity in the control temperature room, which loosens the mesopore walls. In the next section, the drying shrinkage test result confirms the reason, it shows that the shrinkage in M3 was the highest among all other samples. The same phenomenon of reduction in strength was reported by Yosaku Ikeo [44], who investigated different curing conditions of AAMs.

Compressive strength of more than 40 MPa and flexural strength of 6 MPa were achieved using 32% concentrated sodium metasilicate solution, which is much lower than the concentration of sodium silicate solution used in making traditional geopolymer and AAMs [11, 37, 38, 47, 48, 49].

### 3.4 Drying shrinkage

Drying shrinkage of the samples was studied according to [50]. The test was conducted to study the influence of the mixed proportion of the aluminosilicate precursors activated by the AA solution. The AA solution was formed by sodium metasilicate and sodium hydroxide on the drying shrinkage property of the AAM.

**Figure 4:**
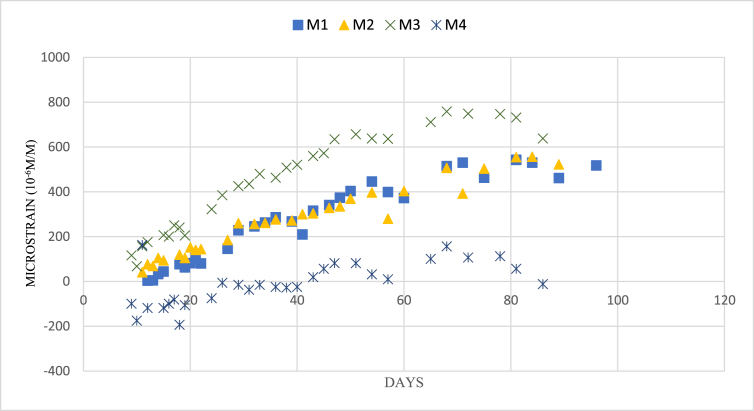
Shrinkage test result.

The M1, M2, and M3 samples had higher amounts of shrinkage compared to M4 due to the presence of the slag and silica fume in their composition, which caused the quick reaction and early loss of water in the mixture. The shrinkage of the samples was increasing until 50 days. After that, it was decreased and then flattened. In contrast, the shrinkage of the 100% fly ash sample was nearly 0 microstrain at the age of around 90 days. This is attributed to the slow reactivity of the precursor [37]. The drying shrinkage of each sample is illustrated in Figure 4.

The shrinkage of all samples was below 0.07%, but it was within the range of allowable shrinkage defined by the ACI 209R, which is 0.05%–0.078% [51]. This may be due to the curing of the samples at 45 °C in the incubator, which conforms to the result of [49].

### 3.5 Setting time

The setting time test was conducted to understand the retarding influence of sucrose on AAMs. The initial and final setting times of P2 and P4 were studied according to JIS R 5201. The mixed proportion of aluminosilicate precursors of M2 and M4 was used to make pastes, which are named P2 and P4, respectively. Table 4 shows the details of each paste. The pastes were studied in the presence and absence of sucrose. The sucrose used in this experiment was 2% of the weight of the precursors of each paste.

**Table 4:** Naming of samples according to the presence and absence of the sucrose.

Paste origin	Without sucrose	With the 2% sucrose
P2	P2S0%	P2S2%
P4	P4S0%	P4S2%

**Figure 5:**
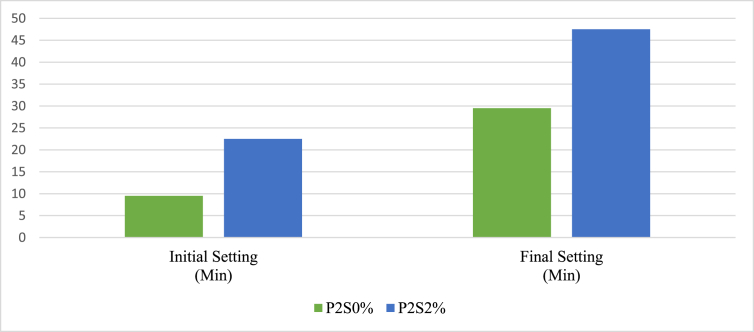
Setting test result of the P2.

**Figure 6:**
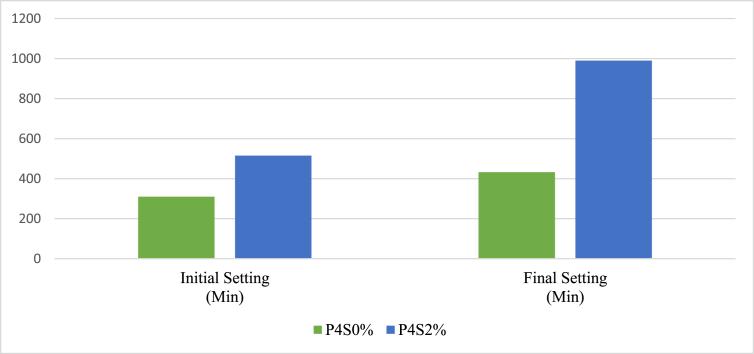
Setting test result of the P4.

The liquid to the binder ratio was kept to 0.19 and 0.17 for the P2 and P4 pastes, respectively. Figures 5 and 6 show the initial and final setting times of P2 and P4 pastes with and without 2% sucrose, respectively. The result shows that sucrose retarded the initial and final setting times of the pastes significantly. The P2 paste without sucrose (P2S0%) had short initial and final setting times of 10 and 30 min, respectively, but adding 2% sucrose increased the paste's initial and final setting time to 23 and 48 min, respectively.

The P4 pastes, P4S0% and P4S2%, followed the same trend: sucrose increased the initial setting time of P4 from 310 to 515 min and the final setting time from 433 min to 990 min.

The initial and final setting time test results of the pastes revealed that the retarding behavior of sucrose can also be observed on AAMs. Many researchers have confirmed its retarding effect on OPC materials in the past [40, 41]. The retarding behavior of an admixture is attributed to the mechanism of adsorption or poisoning of the hydrate surface [52]. Baruere [53] suggested that when monosaccharides and disaccharides convert to saccharinic acids by diluting alkali, these acids have retarding ability through adsorbing onto cement particles. Also, Milestone [54] concluded that sugar and sugar acids adsorb onto the Ca ions that form tri-calcium silicate particles. So, blockage of hydration is occurring when the calcium silicate hydrate nucleation site surface is poisoned by the adsorption of sugar-acid anions. Sugar can be hydrolyzed in strong alkali and its products cover the cement compositions or the cement particles themselves [53, 54]. Therefore, it can be hypothesized that in AAMs, sucrose is first hydrolyzed by alkalis, such as NaOH and sodium silicate, then their product covers the main particles, such as fly ash and slag, for a period, which avoids the reaction or hydration process.

## 4 Conclusion

One-part mixing method was studied by many researchers to make ease in the production of AAMs and geopolymers and to generalize the materials. None of the researchers checked the solubility of the alkaline materials used in the aforementioned mixing method. Therefore, this research studied the dissolution mechanism and solubility of the alkaline materials used in a one-part or dry mixing method. Moreover, the aqueous solution from sodium metasilicate and NaOH was made to overcome workability and rheological problems. Furthermore, the retardation behavior of sucrose on AAM was studied by setting time test. This study concludes as follow:

1) The dissolution of granular Na_2_SiO_3_.5H_2_O increases with an increase in temperature. Total dissolution of the material for making 32% sodium metasilicate solution occurs at a temperature of around 40 °C. If the aforementioned type and concentration of water glass or sodium silicate are changed, then a different temperature of a solvent will be required to totally dissolve the material. Therefore, it is essential to check the solubility of sodium silicate before using it in a one-part mixing method.
2) The alkaline solution formed by the sodium metasilicate is more fluent and smoother. This is due to the lower amount of SiO_2_ and concentration of sodium metasilicate solution. However, the alkaline solution is more sensitive to the lower temperature. Crystallization of the solution can easily happen at a temperature below 30 °C. The same phenomena of crystallization of water glass can also occur while adopting a dry mixing method or one-part mixing method. This crystallization can reduce the reaction process between alkali and aluminum silicate precursors that result in reduction in the quality of the alkali-activated product. To avoid crystallization and to keep the solution smooth and fluent, storing it at a higher temperature is recommended.
3) A compressive strength of around 40 MPa can be achieved by combined fly ash and slag-based AAM activated by a low concentrated sodium metasilicate and NaOH solution.
4) Using sodium metasilicate solution in the two-part mixing method can solve the viscosity problem created by a higher modulus (SiO_2_:Na_2_O = 2) sodium silicate solution.
5) Sucrose retards the reaction process between aluminosilicate precursors and AA solutions in AAM, hence, the setting time is delayed.

## Declarations

### Author contribution statement

Mohammad Idris Rasuli: Conceived and designed the experiments; Performed the experiments; Analyzed and interpreted the data; Wrote the paper.

Yuyun Tajunnisa: Conceived and designed the experiments; Performed the experiments; Analyzed and interpreted the data.

Akifumi Yamamura: Performed the experiments.

Mitsuhiro Shigeishi: Conceived and designed the experiments; Contributed reagents, materials, analysis tools or data.

### Funding statement

This work was supported by the 10.13039/501100012009Hitachi Global Foundation Ref. RS-15, E-1 March 8, 2019, 10.13039/501100004091Kumamoto University, and 10.13039/501100013355Institut Teknologi Sepuluh Nopember.

### Data availability statement

Data related to our research is not publicly available at this time, but will be deposited in a publicly accessible repository after the dissertation, including the contents of this thesis, is reviewed.

### Declaration of interests statement

The authors declare no conflict of interest.

### Additional information

No additional information is available for this paper.
